# Hydroa vacciniforme-like lymphoma of an adult: a case report with review of the literature

**DOI:** 10.1186/1746-1596-8-72

**Published:** 2013-05-01

**Authors:** Mi Wang, Sheng Wang, Qun-Pei Yang, Yan-Mei Liu, Li-Min Gao, Hong Sun, Wei-Ping Liu

**Affiliations:** 1 Department of Dermatovenerology, West China Hospital of Sichuan University, Chengdu, Sichuan, 610041, China; 2 Department of Pathology, West China Hospital of Sichuan University, Chengdu, Sichuan, 610041, China

**Keywords:** Hydroa vacciniforme-like lymphoma, Epstein-Barr virus, Adult

## Abstract

**Virtual slides:**

The virtual slide(s) for this article can be found here: http://www.diagnosticpathology.diagnomx.eu/vs/7644172219178472

Hydroa vacciniforme-like lymphoma (HVL) is a rare type of Epstein-Barr virus (EBV)-positive lymphoma of cytotoxic T-cell or natural killer cell origin that mainly affect children, characterized by a vesicopapular skin eruption that clinically resemble hydroa vacciniforme (HV). In current study, we report an adult patient with the tumor. The patient presented similar morphologic, immunophenotypic and genotypic changes of the disease with that occurred in children, whereas clinically, he showed a prolonged clinical course without hepatosplenomegaly or generalized lymphadenopathy. Whether there are some differences in biologic behavior between children and adults still remains unknown and it is necessary to collect more data to observe and to investigate in the future.

## 

Hydroa vacciniforme-like lymphoma (HVLL) is a cutaneous cytotoxic T-cell or natural killer (NK) cell originated lymphoma occurring in children and adolescents, but not in adults, mainly reported from Asia, Central and South America. In the updated WHO Classification of tumor of Haematopoietic and lymphoid tissues (2008), hydroa vacciniforme-like lymphoma has been recognized as one of the Epstein-Barr virus (EBV)-positive lymphoproliferative disorders of childhood [1]*.* In current study, an adult patient with hydroa vacciniforme-like lymphoma was reported with review of the literature.

## Case history

**Figure 1:**
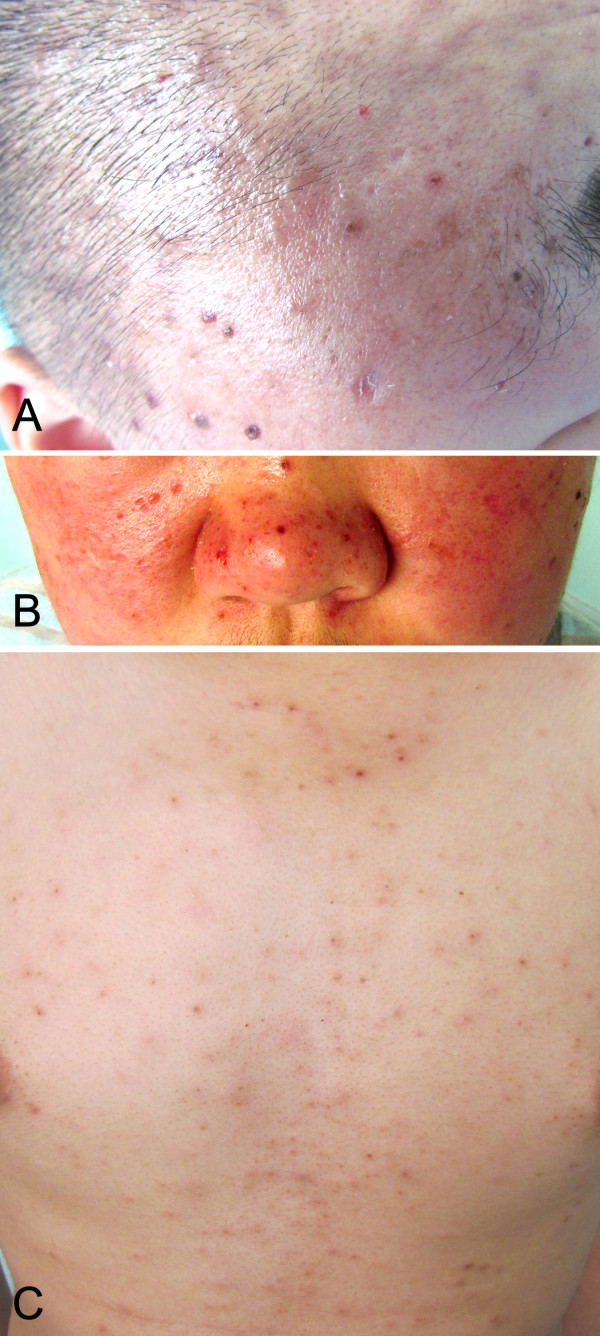
**Skin lesions of hydroa vacciniforme-like lymphoma on the onset.** Papulovascular eruptions on the head (**A**), facial skin around nose (**B**) and thoracic skin (**C**).

A 19-year-old Asian male, complained of repeatedly hyperpyrexia for two years, multiple papules of the head for more than one year, papules and blisters of the face and trunk for eight months (Figure 1). Two years ago, the patient suffered from repeating fever, accompanying with chilly, oral ulcer and night sweat as well. The examinations for tuberculosis showed negative result. His temperature came to normal level after anti-infection therapy for about a week, but relapsed, also accompanying with cough and sputum. “Acute bronchitis caused by salmonella infection” was diagnosed, antibiotics and allopathic treatment were given. The fever was down and the patient was discharged from the hospital. Later on, the patient received anti-tuberculosis therapy for 9 months, in which period his temperature is in the normal level. The patient stopped anti-tuberculosis treatments and had a fever again after three months. More than one year ago, several needle point-sized papules appeared on his vertex, and the patient was treated as “acne folliculitis”, but the skin lesions increased and spread to the whole scalp. Eight months ago, the patient complained of non-pitting edema over his whole face, especially severe in the eyelids. There was no abnormal urination or lower limb edema. Seven months ago, several needle-point-to-millet-sized papules appeared on the face, accompanying with a little itching and blisters. The blisters were Nikolsky’s sign, filled with clear yellow-colored fluid. Once the blisters were ruptured, there leaved depressed cicatrix. The patient was treated with Chinese homeopathic medicine (details unknown), but the fever still kept occurring and the skin lesions spread to the whole face, neck, trunk and both upper limbs, with a pain in distal joints of both hands. Clinically, hydroa vacciniforme-like lymphoma was suspected. Laboratory examinations showed that red blood cell (RBC) 4.28×10^9^/L, white blood cell (WBC) 4.10×10^9^/L, hemoglobin 124 g/L; Lactate dehydrogenase (LDH) 461 IU/L, CD3 subpopulation 83.20%. EBV-DNA level was 1.33E+04 copies/mL detected by real-time quantitative Polymerase Chain Reaction (RT-qPCR). EBV-VCA-IgA was positive, while EBV-EAD-IgG was negative. Type-B ultrasonic examination showed portal vein thickening and splenomegaly. Sputum culture found Pseudomonas aeruginosa. Cervical lymph node biopsy was performed and systemic EBV-positive T-cell lymphoproliferative disease was diagnosed. Clinically, respiratory tract infection was also considered. The protocol of this study was approved by the Institutional Review Board for ethical committee of West China Hospital of Sichuan University, and the study protocol adhered to guidelines by Helsinki Convention.

### Histopathology, immunophenotype, genotype, and EBV status

**Figure 2:**
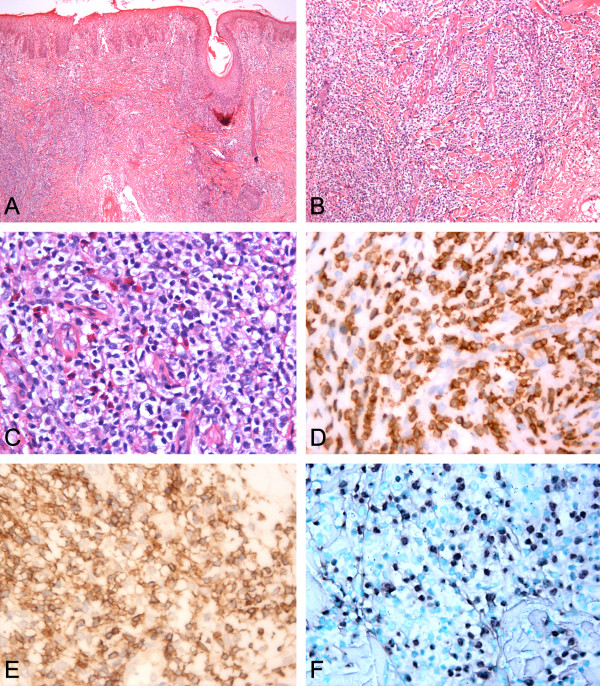
**Morphology changes, Immunohistochemical stain and in situ hybridization for EBV-encoded small RNA (EBER) of hydroa vacciniforme-like lymphoma. A**. The infiltrate is mainly concentrated in the epidermis and dermis, not subcutaneous areas. **B**-**C**. Neoplastic cells are predominantly small without marked atypia. **D**. CD3ϵ positive. **E**. CD2 positive. **F**. EBER positive.

Skin biopsy was performed. It featured an infiltration of a variety of lymphocytes, plasma cells and neutrophils surrounding the labial glands. No clear subepidermal blister was found. In epidermis, a diffuse polymorphic lymphoid infiltrate were found and the infiltrate pattern were mainly perivascular and periadnexal. The infiltrates were prominently composed of small-to-medium-sized atypical lymphocytes with moderate pale stained cytoplasm. The nuclei were oval, round or irregular, with fine chromatin and inconspicuous nucleoli. Mitotic figure was not easy to be found (Figure 2A-C). Some of eosinophils intermingled. Neither necrosis nor erythrophagocytosis was observed. Immunophenotype analysis showed that these lymphocytes were positive for CD3ϵ (Figure 2D), CD2 (Figure 2E), CD7, TIA-1, granzyme B and negative for CD4, CD8, CD20, CD30, CD56, CD79a, CD117, S-100 and Langerin with Ki-67 index being 40%~50%. EBER-positive cells were detected according to *in situ* hybridization for EBV-encoded small RNA (Figure 2F). On the basis of morphology, immunohistochemical stain, and *in situ* hybridization, as well as the clinical menifestations, the diagnosis of hydroa vacciniforme-like lymphoma was established.

### Treatment and follow-up

The patient was treated with Haitangheji, a kind of Chinese homeopathic medicine which is used for immuno-suppression and anti-inflammation, ketotifen which is most commonly used to treat IgE-mediated allergic diseases, and other supportive treatments. α-interferon was used to control the fever. Levofloxacin and sirolimus were used to control fever, but the temperature still kept above 38°C; then prednisone was added. At last, the patient’s temperature fell down to normal level and skin lesions were also gradually disappeared and he was discharged from the hospital. Three month later, the patient died, and no more detail information about the disease was available.

## Discussion

We reported an exceptional case of EBV-associated T/NK-cell lymphoproliferative disorder in an adult who shares similar characteristics of hydroa vacciniforme-like lymphoma in the current WHO category.

EBV-positive T/NK-cell lymphomas recognized by the WHO classification (2008) include extranodal NK/T-cell lymphoma nasal type, aggressive NK-cell leukemia, systemic EBV-positive T-cell lymphoproliferative disorder of childhood, and hydroa vacciniforme-like T-cell lymphoma. All occur more frequently in Asians, and in the Native Americans from Central and South America and Mexico [2]. Except for hydroa vacciniforme-like lymphoma, all are aggressive diseases, with a final fatal outcome in most cases.

Hydroa vacciniforme-like lymphoma (HVLL) has also been called angiocentric cutaneous T-cell lymphoma of childhood [3], and hydroa-like cutaneous T-cell lymphoma [4-6]. Most reports come from Asian area, including Japan, China and Korea [7]. Up to now, limited researches about Hydroa vacciniforme-like lymphoma in adult were reported in the literature due to its rarity.

HVLL is characterized by recurrent papulovesicles which mainly presented in the sun-exposed area, and often accompanied by fever, lymadenopathy, hepatosplenomegaly and increased liver enzyme, and the two year survival rate is 36% [7]. In current report, the patient shares the similar morphologic, immunophenotypic and genotypic changes of HVLL in childhood reported in the literature, whereas clinically, he has a prolonged history (more than three years), and only submendible lymphadenopathy was detected with mild liver enzyme abnormality. Whether there are some differences in biological behavior between children and adult patients still needs necessary investigation in the future.

Hydroa vacciniforme-like lymphoma should be distinguished from a group of T-cell and NK cell lymphoproliferative disorders presenting in the skin. The first is Hydroa vacciniforme (HV). Typical HV is a self-limited skin disease characterized by vesiculopapular eruptions on the sun-exposed regions with vacciniforme scarring. Systemic involvement is absent. Patients with HV-like lymphoproliferative disorders may show some clinical features mimicking typical HV at the onset of the disease [8]. In addition, some cases of severe HV reported in the literature before are actually included in Hydroa vacciniforme-like lymphoma now [7]. The second differential diagnosis is extranodal NK/T-cell lymphoma, nasal type (ENKTCL-N). Unlike HVLL, cutaneous ENKTCL-N usually presents multiple, generalized and severe skin lesions, involving extremities and trunks simultaneously, in sun-exposed and also nonexposed area of the skin. The neoplastic cells infiltrated diffusely in both dermis and subcutis, resulting in a column-like low-power appearance and featured positive for CD56 and usually lack of T-cell receptor gene rearrangement [9]. Furthermore, subcutaneous panniculitis-like T-cell lymphoma (SPTCL) presents with deep subcutaneous nodules rather than vesiculopapalar skin eruptions and is invariably negative for EBV. In addition, primary cutaneous gamma-delta T-cell lymphoma, which is now included in primary cutaneous peripheral T-cell lymphoma, not otherwise specified is extremely rare and is also negative for EBV.

With regard to the treatment of the tumor, Interferon-α (IFN-α) is the most commonly selected whose major side effects are fever and cytopenia. Corticosteroid is usually used to control the symptoms, but it is not suitable to be used persistently. Chemotherapy is uncommonly used, because only 30% of patients may have partial response. Xu reported six Chinese cases of childhood, in which, four patients received IFN-α, and two of them also had chemotherapy (the regimen was unknown) [8]. In current report, the patient was treated by IFN-α and corticosteroid. Although the effect is not so ideal, the progression is relatively slow. The prognosis-related factor is unclear right now. According to the limited reports in the literature, the clinical course may be indolent in patients with the NK-cell phenotype; whereas cases with T-cell phenotype are characterized by intermittent fever and hepatosplenomegaly. High titers of EBV-related antibodies in the serum often predict a progressive clinical course and poor prognosis.

## Consent

Written informed consent was obtained from the patient for publication of this case report and any accompanying images. A copy of the written consent is available for review by the Editor-in-Chief of this journal.

## Competing interests

The authors declare that they have no competing interests.

## Authors’ contributions

MW, SW and QPY conducted data analysis and helped to draft the manuscript. YML, LMG and HS carried out the immunohistochemical staining and molecular genetic studies. WPL wrote the manuscript and supervised throughout the study. All authors read and approved the final manuscript.
